# Follicular Klotho in the Ovarian Microenvironment: Exploring Its Role in IVF Outcome Prediction

**DOI:** 10.3390/medicina62010139

**Published:** 2026-01-09

**Authors:** Mehmet Alican Sapmaz, Sait Erbey, Murat Polat, Selin Yıldız, İnci Kahyaoğlu, Ömer Osman Eroğlu, Emine Utlu Özen, Ayfer Bakır

**Affiliations:** 1 Department of Obstetrics and Gynecology, Etlik City Hospital, 06170 Ankara, Türkiye; saiterbey@gmail.com (S.E.); dr.muratpolat@hotmail.com (M.P.); mdincikahyaoglu@gmail.com (İ.K.); omerosmaneroglu@gmail.com (Ö.O.E.); 2 Department of Clinical Biochemistry, Etlik City Hospital, 06170 Ankara, Türkiye; slnnnyldzzz89@gmail.com; 3 IVF Center, Laboratory of Embryology, Etlik City Hospital, 06170 Ankara, Türkiye; eminetluozen@gmail.com; 4 Department of Medical Microbiology, Etlik City Hospital, 06170 Ankara, Türkiye; dr.ayfer.bakir@gmail.com

**Keywords:** klotho protein, follicular fluid, in vitro fertilization, implantation, clinical pregnancy

## Abstract

*Background and Objectives:* Klotho (KL) is a multifunctional protein involved in reproductive physiology; however, its precise role in ovarian reserve and in vitro fertilization (IVF) outcomes remains unclear. This study aimed to evaluate the relationship between follicular fluid KL levels, ovarian reserve markers, and key IVF success parameters. *Materials and Methods:* This prospective study included a total of 150 women undergoing IVF, of whom 82 had diminished ovarian reserve (DOR) and 68 had normal ovarian reserve (NOR). All participants underwent controlled ovarian stimulation using a standard antagonist protocol. During oocyte pick-up (OPU), the first aspirated follicular fluid sample was collected, processed, and analyzed for KL concentrations using a Human Klotho ELISA kit. Hormonal profiles, ovarian reserve markers, and IVF outcomes were compared between groups. *Results:* Follicular fluid KL levels were significantly lower in the DOR group compared with the NOR group (117.07 ± 28.88 pg/mL vs. 266.13 ± 58.29 pg/mL; *p* < 0.001). Anti-Müllerian hormone (AMH) levels were reduced, whereas follicle-stimulating hormone (FSH), luteinizing hormone (LH), and estradiol (E2) levels were significantly higher in the DOR group (all *p* < 0.001). Implantation and clinical pregnancy rates were also significantly lower in the DOR group compared with the NOR group (*p* < 0.001 and *p* = 0.003, respectively). KL levels showed a strong positive correlation with the number of fertilized oocytes in both groups (DOR: r = 0.690; NOR: r = 0.552). Each one-unit increase in KL was associated with a 3.7% increase in implantation probability and a 3.2% increase in clinical pregnancy probability in the DOR group, and with corresponding increases of 4.4% and 1.2% in the NOR group (all *p* < 0.05). *Conclusions:* This study demonstrates significant associations between follicular fluid KL levels and fertilization, implantation, and clinical pregnancy outcomes. These associations appear to be more pronounced than those observed with traditional ovarian reserve markers such as AMH and antral follicle count. Reduced KL levels are associated with fewer fertilized oocytes, whereas higher KL concentrations correspond to increased implantation and clinical pregnancy probabilities. Nevertheless, similar to other non-invasive biomarkers, current evidence is insufficient to support routine clinical use of KL. Large-scale, well-designed, multicenter studies are therefore required to validate its clinical relevance and to determine whether KL can serve as a reliable and practical predictor of IVF success.

## 1. Introduction

The Klotho (KL) gene, discovered in 1997, is located on the long arm of chromosome 13 (13q) and has been identified as a key inhibitory gene in the aging process and the development of age-related diseases [1]. In humans, the gene consists of five exons and four introns and, through alternative mRNA splicing, encodes two distinct KL protein isoforms: a membrane-bound form and a soluble form. The membrane-bound form functions as a co-receptor with the fibroblast growth factor receptor, mediating the activity of fibroblast growth factor 23 in phosphate and vitamin D metabolism, whereas the soluble form regulates ion transport [2,3], Wnt signaling pathways [4], antioxidant defense, anti-aging mechanisms, and calcium and parathyroid hormone homeostasis [5]. The KL protein is primarily synthesized in the renal tubular epithelial cells and choroid plexus, with lower expression levels observed in the pituitary gland, placenta, skeletal muscle, urinary bladder, pancreas, testis, ovary, and colon [6,7,8].

KL regulates oxidative stress and cellular senescence primarily through the endothelial nitric oxide synthase [9] and insulin-like growth factor-1 (IGF-1) [10] signaling pathways. Given that DNA methylation plays a pivotal role in oncogenesis, it has been proposed that KL exerts tumor-suppressive effects by inhibiting IGF-1 signaling, particularly in cervical and breast cancers [11,12]. Furthermore, the promoter methylation of the KL gene has been detected in approximately 46% of gastric cancer patients, supporting the hypothesis that KL may represent a novel epigenetically regulated tumor suppressor gene [13]. Although most researchers have focused on the systemic and oncogenic roles of Klotho, growing evidence indicates that this multifunctional protein also plays a crucial role in reproductive physiology and ovarian aging.

The KL protein is highly expressed in the suprachiasmatic nucleus of the hypothalamus, and its deficiency has been shown to disrupt the hypothalamic-pituitary-ovarian axis (HPO), leading to the decreased secretion of follicle-stimulating hormone (FSH) and luteinizing hormone (LH), follicular arrest, gonadal atrophy, and ultimately infertility [14]. Animal models of BRCA mutation-related premature ovarian failure (POF) have exhibited significantly reduced serum KL concentrations, suggesting that KL deficiency may contribute to the pathophysiology of ovarian insufficiency and reproductive aging [15,16,17,18]. Collectively, these findings indicate that KL may act as a molecular link between oxidative stress, cellular senescence, and diminished ovarian function.

The PI3K/Akt pathway is essential for oocyte growth and follicular development, with FOXO3 mediating granulosa cell proliferation and oxidative stress regulation. Impaired PI3K/Akt FOXO3 signaling, characterized by reduced superoxide dismutase (SOD) and increased reactive oxygen species (ROS), has been implicated in POF, and similar dysregulation disrupts primordial follicle formation in humans [19,20]. KL suppresses PI3K/Akt activation, thereby enhancing FOXO3 function, promoting MnSOD expression, and limiting oxidative damage, whereas KL deficiency accelerates ovarian aging through oxidative imbalance [21]. KL also influences Wnt/β-catenin signaling, and its loss impairs granulosa cell function and folliculogenesis [22,23]. Together, these findings indicate that KL modulates ovarian aging through multiple interconnected pathways; however, its presence and clinical relevance within human follicular fluid (FF) remain largely undefined.

FF reflects the biochemical microenvironment of the developing oocyte and serves as a valuable source of noninvasive markers of oocyte and embryo competence. Peptidomic analyses, including the mass spectrometry–based study by Chen et al., have identified FF biomarker panels capable of predicting fertilization outcomes with high accuracy, underscoring the diagnostic potential of FF [24]. The close interplay between FF and the cumulus–oocyte complex further highlights its critical role in oocyte maturation and developmental competence [25]. Nevertheless, despite these advances, clinically reliable FF biomarkers for assessing ovarian reserve and predicting IVF success remain limited.

Although FF analysis offers valuable mechanistic insights, routine measurement of individual FF proteins has not yet yielded practical tools for predicting IVF outcomes. With advances in genomic technologies, targeted protein modulation, and recombinant therapeutics, proteins such as KL may eventually serve not only as biomarkers but also as potential modulators of ovarian function. Accordingly, this study investigated FF KL concentrations and their associations with ovarian reserve, oocyte quality, fertilized oocytes, and early pregnancy outcomes in women undergoing IVF.

## 2. Materials and Methods

### 2.1. Study Design and Ethical Approval

This prospective cohort study was conducted at the IVF Clinic of the Department of Obstetrics and Gynecology, Etlik City Hospital, Ankara, Türkiye, and ethical approval for this study was obtained on 26 March 2025 under the protocol code AEŞH-BADEK-2025-0582. Women between 23 and 39 years of age who provided written informed consent were included, and they consisted of those who presented to our center between April and November 2025.

### 2.2. Study Population and Grouping

A total of 150 patients were enrolled. Of these, 82 women were diagnosed with DORs and 68 had NORs, with infertility attributed to male or tubal factors.

The diagnosis of a DOR was based on the AMH, FSH, and AFC values [26,27,28]. Patients were classified as having a DOR if all the following criteria were met: a total bilateral AFC ≤ 7 and AMH ≤ 1.1 ng/mL or basal FSH ≥ 10 IU/L, measured on the second or third day of a spontaneous menstrual cycle. The NOR group included women undergoing IVF for male or tubal factor infertility, with AMH ≥ 2 ng/mL, an AFC > 7, basal FSH < 10 IU/L, and E2 < 80 pg/mL.

Inclusion Criteria: Women were included if they were between 23 and 39 years of age, provided written informed consent, underwent IVF for male or tubal factor infertility, and had regular menstrual cycles with a baseline hormonal assessment performed on days 2–3 of the cycle. Additionally, only patients who voluntarily agreed to participate in this study and completed and signed the informed consent form were included.

Exclusion Criteria: Women were excluded if they had malignancy, autoimmune disease, or any severe endocrine or metabolic disorders, such as diabetes mellitus or hypothyroidism. Those with chronic inflammatory diseases, a history of hormonal medication use, or prior ovarian surgery were also excluded. Additional exclusion criteria included the use of donor oocytes and a diagnosis of polycystic ovary syndrome (PCOS), polycystic ovarian morphology, or endometriosis. A clinical pregnancy was defined as the presence of at least one gestational sac on ultrasonography and a positive β-hCG result. Furthermore, patients who did not wish to voluntarily participate in this study or who did not complete and sign the informed consent form were not included.

Implantation was defined at the patient level as the presence of at least one gestational sac detected by transvaginal ultrasonography following embryo transfer.

### 2.3. Ovarian Stimulation Protocol

Controlled ovarian stimulation was performed using a uniform protocol in all patients. Recombinant FSH (rec-FSH) was administered at daily doses of 225–300 IU (subcutaneously), adjusted according to the ovarian reserve parameters and body mass index (BMI). A fixed GnRH antagonist protocol was used, with the antagonist introduced on day 6 of stimulation and continued until the trigger day. All cycles were monitored via transvaginal ultrasonography and serum LH, estrogen, and progesterone levels. Ovulation was triggered with human chorionic gonadotropin (hCG) when at least three follicles measured ≥ 17 mm, and oocyte retrieval (OPU) was performed 35–36 h later.

### 2.4. Follicular Fluid Collection and Storage

During OPU, the first FF aspirate was collected without flushing, confirmed to contain an oocyte, and transferred into sterile, conical Falcon tubes. Samples were maintained at 4 °C for 4–6 h and were then centrifuged at 1000× *g* for 10 min at room temperature to remove erythrocytes and leukocytes. The supernatant was transferred into cryovial tubes using sterile pipettes and stored at −80 °C until analysis.

### 2.5. Measurement of Klotho Protein Concentrations

The FF soluble Klotho (sKL) protein concentrations were measured by a biochemistry specialist using a quantitative sandwich enzyme-linked immuno-sorbent assay (ELISA) according to the manufacturer’s instructions. A Human Klotho ELISA Kit (BT LAB, Cat. No: E278111u; Bioassay Technology Laboratory, Shanghai, China) that specifically detects the circulating soluble form of Klotho was used. The standard curve range was 5–1000 pg/mL, and the limit of detection (LOD) was 2.47 pg/mL.

The Klotho concentrations were calculated based on the absorbance values using the standard calibration curve. All samples were analyzed in duplicate, and the mean values are reported. The intra-assay coefficient of variation (CV) was <8%, and the inter-assay CV was <10%. Samples were stored at −80 °C before analysis and thawed only once. All analyses were performed in accordance with the manufacturer’s protocol and quality control standards.

### 2.6. Statistical Analysis

All statistical analyses were performed using IBM SPSS Statistics version 26.0 (IBM Corp., Armonk, NY, USA). The distribution of continuous variables was assessed using skewness–kurtosis coefficients and the Shapiro–Wilk test, and all variables demonstrated normal distribution within acceptable limits (skewness and kurtosis between −3 and +3) (Table 1). Continuous variables were summarized as means ± standard deviations (SDs), whereas categorical variables were reported as frequencies and percentages.

Comparisons between the DOR and NOR groups were conducted using an independent-samples *t*-test for continuous variables and a chi-square or Fisher’s exact test for categorical variables, as appropriate.

The relationships between FF Klotho concentrations and reproductive parameters (AMH, FSH, LH, E2, AFC, number of oocytes retrieved, fertilized oocytes) were evaluated using Pearson correlation analysis. Fertilization outcome was expressed as the fertilization rate, calculated as the percentage of fertilized oocytes relative to the total number of retrieved oocytes for each patient.

To identify independent predictors of implantation and clinical pregnancy, multivariable logistic regression analyses were performed separately for the DOR and NOR groups. Variables included in the regression models were Klotho level, AMH, FSH, and AFC. The selection of covariates was based on biological relevance and to avoid model overfitting given the sample size. Age and BMI were not included in the multivariable models because these parameters did not differ significantly between the DOR and NOR groups and showed no significant association with implantation or clinical pregnancy outcomes in univariable analyses. Ovarian stimulation protocol–related variables were not included, as a uniform stimulation protocol was applied to all participants. Odds ratios (ORs) with 95% confidence intervals (CIs) and *p*-values were reported. Statistical significance was defined as *p* < 0.05 (two-tailed). The number of embryos transferred was not included as a covariate in the regression models; therefore, implantation outcomes were analyzed on a per-patient basis.

## 3. Results

### 3.1. Comparison of Hormonal and Clinical Parameters

The mean Klotho level (pg/mL) was significantly lower in women with DORs than that in the NOR group (*p* < 0.001). No statistically significant difference was observed between the groups in terms of age or BMI (*p* = 0.997 and *p* = 0.638, respectively).

The serum anti-Müllerian hormone (AMH) levels were markedly reduced in the DOR group, whereas the FSH levels were significantly higher compared with those of the NORs (both *p* < 0.001). Similarly, the LH and estradiol (E2) concentrations were significantly elevated in the DOR group (both *p* < 0.001).

In contrast, the antral follicle count (AFC), number of oocytes retrieved, and fertilized oocytes were all significantly lower among patients with DORs compared with the NORs (all *p* < 0.001).

A detailed comparison of the hormonal and clinical parameters between groups is presented in Table 2.

### 3.2. Implantation and Clinical Pregnancy Outcomes

The rates of patients without implantation were 37.8% (n = 31) in the DOR group and 8.8% (n = 6) in the NOR group.

The proportions of cases with successful implantation were 62.2% (n = 51) in the DOR group and 91.2% (n = 62) in the NOR group (*p* < 0.001).

The rates of patients without clinical pregnancy were 69.5% (n = 57) in the DOR group and 45.6% (n = 31) in the NOR group, while those of patients with clinical pregnancy were 30.5% (n = 25) in the DOR group and 54.4% (n = 37) in the NOR group (*p* = 0.003) (Table 3).

### 3.3. Klotho Levels and Related Variables

In the DOR group, a strong positive correlation was observed between Klotho levels and fertilization (r = 0.690). AMH showed a moderate positive correlation with menstrual cycle day (r = 0.420) and a weak positive correlation with FSH (IU/L) (r = 0.255). AFC demonstrated a moderate positive correlation with menstrual cycle day (r = 0.361), as well as weak positive correlations with LH (r = 0.230) and E2 (r = 0.264). Additionally, AFC exhibited a moderate positive correlation with the number of oocytes retrieved (r = 0.578).

In the NOR group, LH demonstrated a weak positive correlation (r = 0.261), and a moderate positive correlation was found between Klotho levels and fertilization (r = 0.552). AMH showed moderate positive correlations with LH (r = 0.410) and E2 (r = 0.371), while a moderate negative correlation was observed with the number of oocytes retrieved (r = −0.438) (Table 4).

### 3.4. Implantation Outcomes and Group-Specific Variable Effects

In the DOR group, Klotho level was the only variable that positively influenced the likelihood of implantation (OR = 1.037, *p* = 0.004). Accordingly, higher Klotho concentrations were associated with an increased probability of implantation. When expressed per clinically meaningful increments, a 10 pg/mL increase in follicular fluid Klotho level corresponded to an approximately 44% increase in implantation probability in the DOR group (OR ≈ 1.44), while a 50 pg/mL increase was associated with an approximately fivefold higher likelihood of implantation. Similarly, in the NOR group, Klotho level was the sole variable demonstrating a significant positive effect on implantation (OR = 1.044, *p* = 0.041), indicating that implantation likelihood increased as Klotho levels rose (Table 5).

### 3.5. Clinical Pregnancy Outcomes and Group-Specific Variable Effects

In the DOR group, Klotho level was the only variable that positively influenced the likelihood of achieving a clinical pregnancy (OR = 1.032, *p* = 0.002). Accordingly, higher Klotho concentrations were associated with an increased probability of clinical pregnancy. When expressed per clinically meaningful increments, a 10 pg/mL increase in follicular fluid Klotho level corresponded to a substantial increase in the probability of clinical pregnancy, with larger increments translating into progressively higher odds. Similarly, in the NOR group, Klotho level was the sole variable demonstrating a significant positive effect on clinical pregnancy (OR = 1.012, *p* = 0.020), indicating that the likelihood of achieving a clinical pregnancy increased as Klotho levels rose (Table 6).

## 4. Discussion

In this study, we examined the relevance of FF Klotho levels to ovarian function, ovarian reserve, and IVF outcomes. Follicular KL concentrations were significantly lower in women with DOR compared with those with NOR undergoing IVF for male or tubal factor infertility. Importantly, higher KL levels were associated with increased fertilization, implantation, and clinical pregnancy rates, and these associations were stronger than those observed with established ovarian reserve markers such as AMH and AFC. Although these findings do not establish KL as a predictive marker on its own, they suggest that follicular KL may better reflect aspects of ovarian reserve status and reproductive potential related to follicular competence. Collectively, these findings indicate that KL may, in the future, serve as a complementary and correlative biomarker reflecting follicular competence and the local ovarian microenvironment, rather than a replacement for established ovarian reserve markers such as AMH and AFC. Accordingly, follicular fluid KL appears to capture aspects of the local follicular microenvironment and oocyte competence that are not fully reflected by systemic ovarian reserve markers. In addition, although the regression models were constructed using continuous KL values, expressing the effect size per clinically meaningful increments highlights that even moderate increases in follicular KL concentration may translate into substantial differences in implantation and clinical pregnancy probabilities.

The success of IVF treatment holds significant individual and societal importance in the management of infertility. However, despite technological advancements, implantation rates remain suboptimal, and the current methods for assessing embryo and endometrial quality are inadequate for accurately predicting treatment outcomes. Although AMH levels are widely used to estimate the ovarian response to gonadotropin stimulation, their predictive accuracy for pregnancy outcomes is limited by multiple factors, including age, etiology, and the duration of infertility [29,30]. Gomez et al. [31] reported that even extremely low AMH levels do not preclude successful conception, emphasizing that discontinuation of IVF therapy should not rely solely on AMH concentrations. Similarly, in a global survey conducted by Christianson et al. [32] evaluating the impact of AFC on IVF outcomes, most clinicians reported routinely using AFC in clinical practice and tailoring IVF protocols; accordingly, however, the majority did not consider AFC to be the most reliable predictor of ongoing pregnancy. Another study found that although both AMH and AFC correlate with live birth rates, their combined assessment does not improve predictive accuracy, and AMH alone provides limited prognostic value [33]. In our study, follicular Klotho levels demonstrated stronger correlations with fertilization, implantation, and clinical pregnancy than AMH or AFC, suggesting that KL may, in the future, serve as an alternative biomarker for evaluating ovarian reserve, although further validation is required. It should also be acknowledged that measuring KL in FF requires sampling obtained during oocyte retrieval, which is inherently more invasive than serum AMH testing or ultrasound-based AFC assessment. However, because FF is routinely collected during standard IVF cycles without additional intervention for the patient, its analysis may still offer a practical opportunity for generating biologically meaningful data. The differences observed among correlation analyses can be attributed to several methodological and biological factors. First, analyses were stratified according to ovarian reserve status (DOR vs. NOR), resulting in heterogeneous endocrine and follicular microenvironments that may differentially modulate the association between KL levels and reproductive parameters. Second, correlation analyses were performed using continuous variables and unadjusted models, whereas implantation and clinical pregnancy outcomes were evaluated using multivariable logistic regression, which accounts for the combined effects of multiple predictors. Finally, fertilization was expressed as a cycle-level outcome, while implantation and pregnancy represent downstream, multifactorial clinical endpoints. Together, these factors may explain the observed variability in correlation strength across different parameters and analytical approaches.

Recent studies have revealed that the KL gene is expressed within the female hy-HPO axis and may contribute to reproductive endocrine disorders, such as PCOS and POF [8,34]. Experimental studies in animal models have shown that healthy mice possess abundant KL expression localized to the oocyte membranes of mature follicles, whereas Klotho-deficient mice lack mature follicles and display atrophic reproductive organs and infertility [6]. Similarly, Klotho-deficient mice exhibit praxis dysfunction characterized by reduced FSH and LH secretion, follicular arrest, gonadal atrophy, and infertility [25]. In cyclophosphamide-induced POF mouse models, the granulosa cell KL expression was markedly decreased, suggesting that KL acts as a protective factor against follicular depletion and ovarian failure [35].

In agreement with these findings, a human study reported that granulosa cells’ KL expression in the FF of women with DORs was significantly lower than that of controls [36]. Consistent with previous evidence, our results also demonstrated that the FF KL protein levels were markedly reduced in IVF patients with DORs. Importantly, patients with higher KL concentrations showed significantly improved fertilization, implantation, and clinical pregnancy outcomes.

In contrast, Ye et al. [37] observed that the KL levels in both FF and granulosa cells were elevated in women with hyperandrogenic PCOS, suggesting an association with apoptotic and inflammatory gene regulation. The follicular KL levels exhibited a negative correlation with the number of mature oocytes but exhibited positive correlations with the serum testosterone, LH, LH/FSH ratio, menstrual cycle length, and AFC. Additional studies involving both PCOS patients and rat models have confirmed the significant upregulation of the KL expression in granulosa cells, accompanied by increased caspase-3 activity, a key mediator of apoptosis [38]. Moreover, silencing of the KL gene in granulosa cells from PCOS patients reduced the caspase-3 activity, supporting the notion that excessive KL expression may contribute to granulosa cell apoptosis. Based on this evidence, KL inhibition has been proposed as a potential therapeutic approach in PCOS management. Song et al. [39] further suggested that KL plays a critical role in PCOS onset and progression, serving as a potential biomarker for oocyte quality and IVF outcome evaluation. Conversely, in our study involving women with DORs, the FF KL levels were significantly lower than those in the NOR group, suggesting that KL may exhibit distinct modulatory patterns under different pathophysiological conditions, such as PCOS and DOR, indicating the context-dependent role of this protein in ovarian physiology.

One of the major strengths of our study lies in its prospective design, which minimized recall bias, enabled systematic monitoring of clinical and biochemical parameters, and allowed for a more reliable evaluation of associations between follicular fluid Klotho levels and reproductive outcomes. While previous human and animal studies have demonstrated Klotho expression in granulosa cells and follicular fluid, these investigations have largely focused on expression patterns, experimental models, or limited clinical correlations. In contrast, the present study extends the existing literature by prospectively evaluating follicular fluid Klotho concentrations in a well-characterized IVF cohort with diminished ovarian reserve and systematically relating these levels to fertilization, implantation, and clinical pregnancy outcomes. By demonstrating robust associations between follicular fluid Klotho levels and multiple clinically relevant IVF endpoints, our findings provide incremental clinical insight into the potential role of Klotho as a correlative marker of follicular competence and treatment response within this specific patient population. These observations highlight the potential value of KL as a biochemical indicator of the oocyte quality and ovarian function and suggest that KL may serve as a promising biomarker in assisted reproductive technologies.

However, this study has several limitations that should be considered. The relatively small sample size may restrict the generalizability of the findings. In addition, we only measured the FF KL levels and did not assess the serum concentrations, granulosa cell expression, or potential genetic or epigenetic alterations, which limits the ability to fully interpret the biological role of Klotho. Moreover, the lack of distinction between membrane-bound and soluble KL isoforms may also limit mechanistic interpretation, and the absence of a comparative evaluation with oxidative stress markers or other related biomarkers restricts the assessment of the independent Klotho effects. Although this study was not designed to investigate mechanistic pathways, existing experimental evidence suggests that Klotho modulates ovarian function through oxidative stress regulation, PI3K/Akt–FOXO3 signaling, and inflammatory pathways. Future studies incorporating oxidative stress markers, inflammatory mediators, and cell-specific localization of KL isoforms in granulosa and cumulus cells would be valuable to further elucidate the biological mechanisms underlying the observed clinical associations. One important limitation of this study is that implantation was defined on a per-patient basis rather than per embryo. The number of embryos transferred was not included as a covariate in the regression analyses. Since embryo number is a known determinant of implantation probability, this may have introduced residual confounding. Therefore, the observed associations between follicular fluid Klotho levels and implantation outcomes should be interpreted as correlative rather than causal. Although additional clinical covariates such as age, BMI, and stimulation-related parameters may influence IVF outcomes, these variables were not included in the multivariable models because they were homogeneous across groups or standardized by study design. Finally, we did not evaluate the potential modifying effects of ovarian stimulation protocols or fertilization methods on the KL expression and oxidative stress, which may represent additional confounding variables.

## 5. Conclusions

In conclusion, this study offers new insight into the potential role of FF Klotho in human reproductive biology. KL may influence ovarian physiology through mechanisms related to cellular aging, oxidative stress regulation, and follicular microenvironment homeostasis, positioning it as a biologically meaningful indicator of reproductive potential. Our findings suggest that follicular KL may serve as a correlative biomarker of follicular competence and treatment response, complementing rather than replacing established ovarian reserve markers. Accordingly, follicular KL levels may, in the future, serve as a useful marker for predicting IVF success and as a complementary parameter for assessing ovarian reserve alongside existing biomarkers. Nevertheless, larger multicenter prospective studies are required to elucidate the molecular pathways through which KL affects ovarian function and to determine its clinical relevance. As advances in genomic modulation and targeted protein therapies continue to evolve, KL may also emerge as a potential therapeutic target within reproductive medicine.

## Figures and Tables

**Table 1:** Skewness and kurtosis values of continuous variables.

Parameter	Skewness	Kurtosis
Klotho Level (pg/mL)	0.628	−0.781
Age (years)	0.059	−1.014
BMI (kg/m^2^)	0.760	0.476
Menstrual Cycle (days)	−0.117	−0.371
AMH (ng/mL)	0.509	−1.108
FSH (IU/L)	−0.018	−1.156
LH (IU/L)	0.608	−0.348
E2 (pg/mL)	0.238	−0.817
AFC	0.423	−1.015
Oocytes Retrieved	0.360	−1.469
Fertilization	0.985	0.114

Abbreviations: BMI, body mass index; AMH, anti-Müllerian hormone; FSH, follicle-stimulating hormone; LH, luteinizing hormone; E2, estradiol; AFC, antral follicle count. Skewness and kurtosis values within the range of ±3 indicate normal data distribution.

**Table 2:** Comparison of hormonal and clinical parameters between DOR and NOR groups.

Parameter	DOR (n = 82) Mean ± SD	NOR (n = 68) Mean ± SD	*p* Value
Klotho Level (pg/mL)	117.07 ± 28.88	266.13 ± 58.29	<0.001
Age (years)	30.59 ± 4.3	30.59 ± 4.63	0.997
BMI (kg/m^2^)	21.35 ± 2.06	21.19 ± 2.21	0.638
AMH (ng/mL)	0.53 ± 0.27	2.46 ± 0.61	<0.001
FSH (IU/L)	15.31 ± 2.51	7.74 ± 2.39	<0.001
LH (IU/L)	3.51 ± 1.06	4.72 ± 1.1	<0.001
E2 (pg/mL)	110.93 ± 25.11	62.61 ± 19.46	<0.001
AFC	3.3 ± 1.1	10.93 ± 2.36	<0.001
Oocytes Retrieved	3.31 ± 1.09	13.25 ± 2.19	<0.001
Fertilized Oocytes	2.35 ± 0.88	7.65 ± 2.75	<0.001

Values are expressed as means ± standard deviations (SDs). An independent-sample *t*-test was used. *p* < 0.05 was considered statistically significant. Abbreviations: DOR, diminished ovarian reserve; NOR, normal ovarian reserve; BMI, body mass index; AMH, anti-Müllerian hormone; FSH, follicle-stimulating hormone; LH, luteinizing hormone; E2, estradiol; AFC, antral follicle count.

**Table 3:** Implantation and clinical pregnancy statuses between groups.

Parameter	DOR Group (n = 82) n (%)	NOR Group (n = 68) n (%)	*p* Value
Implantation-Negative	31(37.8)	6(8.8)	<0.001
Implantation-Positive	51(62.2)	62(91.2)	
Clinical Pregnancy-Negative	57(69.5)	31(45.6)	0.003
Clinical Pregnancy-Positive	25(30.5)	37(54.4)	

Abbreviations: DOR, diminished ovarian reserve; NOR, normal ovarian reserve; N, total number of participants; n, number within subgroup; %, percentage.

**Table 4:** Klotho levels and related variables.

		DOR			NOR		
		Klotho Level (pg/mL)	AMH (ng/mL)	AFC	Klotho Level (pg/mL)	AMH (ng/mL)	AFC
Menstrual Cycle (Day)	r	0.198	0.420	0.361	0.030	−0.235	0.038
	*p*	0.074	0.000	0.001	0.806	0.053	0.761
FSH (IU/L)	r	0.045	0.255	0.168	0.075	−0.093	0.181
	*p*	0.685	0.021	0.132	0.546	0.453	0.140
LH(IU/L)	r	0.090	−0.077	0.230	0.261	0.410	−0.168
	*p*	0.420	0.492	0.037	0.031	0.001	0.172
E2 (pg/mL)	r	0.131	−0.033	0.264	0.201	0.371	0.063
	*p*	0.241	0.769	0.017	0.100	0.002	0.612
Oocytes Retrieved	r	−0.045	−0.054	0.578	0.102	−0.438	0.153
	*p*	0.686	0.629	0.000	0.409	0.000	0.212
Fertilization	r	0.690	−0.018	0.128	0.552	−0.171	0.230
	*p*	0.000	0.871	0.250	0.000	0.163	0.059

Abbreviations: DOR, diminished ovarian reserve; NOR, normal ovarian reserve; AMH, anti-Müllerian hormone; AFC, antral follicle count; E2, estradiol; FSH, follicle-stimulating hormone; LH, luteinizing hormone; r, Pearson correlation coefficient; *p*, *p*-value.

**Table 5:** Factors influencing implantation outcomes in groups.

Group	Dependent Variable	Independent Variable	B	S.E.	Wald	*p*	OR
DOR	Implantation outcome	Klotho Level (pg/mL)	0.037	0.013	8.195	0.004	1.037
		AMH (ng/mL)	−0.622	0.986	0.398	0.528	0.537
		FSH (IU/L)	0.237	0.127	3.483	0.062	1.268
		AFC	0.316	0.180	3.075	0.080	1.371
NOR	Implantation outcome	Klotho Level (pg/mL)	0.043	0.021	4.180	0.041	1.044
		AMH (ng/mL)	−2.460	1.306	3.545	0.060	0.085
		FSH (IU/L)	−0.343	0.289	1.407	0.236	0.710
		AFC	−0.301	0.287	1.100	0.294	0.740

Abbreviations: DOR, diminished ovarian reserve; NOR, normal ovarian reserve; AMH, anti-Müllerian hormone; FSH, follicle-stimulating hormone; AFC, antral follicle count; B, regression coefficient; S.E., standard error; OR, odds ratio. Odds ratios are presented per 1 pg/mL increase in follicular fluid Klotho level. For clinical interpretability, higher-order increments are described in the Results section.

**Table 6:** Factors influencing clinical pregnancy outcomes in groups.

Group	Dependent Variable	Independent Variable	B	S.E.	Wald	*p*	OR
DOR	Clinical Pregnancy	Klotho Level (pg/mL)	0.031	0.010	9.382	0.002	1.032
		AMH (ng/mL)	−1.321	1.073	1.517	0.218	0.267
		FSH (IU/L)	0.202	0.112	3.247	0.072	1.224
		AFC	0.025	0.192	0.017	0.897	1.025
NOR	Clinical Pregnancy	Klotho Level (pg/mL)	0.012	0.005	5.390	0.020	1.012
		AMH (ng/mL)	−0.469	0.461	1.036	0.309	0.626
		FSH (IU/L)	−0.232	0.132	3.090	0.079	0.793
		AFC	0.180	0.123	2.138	0.144	1.197

Abbreviations: DOR, diminished ovarian reserve; AMH, anti-Müllerian hormone; FSH, follicle-stimulating hormone; AFC, antral follicle count; B, regression coefficient; S.E., standard error; OR, odds ratio. Odds ratios are presented per 1 pg/mL increase in follicular fluid Klotho level. For clinical interpretability, higher-order increments are described in the Results section.

## Data Availability

The data presented in this study are available in the article. Further inquiries can be directed to the corresponding authors.
